# A Hybrid Optimization Algorithm for Enhancing Transportation and Logistics Scheduling in IoT-Enabled Supply Chains

**DOI:** 10.3390/s26030932

**Published:** 2026-02-01

**Authors:** Alaa Abdalqahar Jihad, Ahmed Subhi Abdalkafor, Esam Taha Yassen, Omar A. Aldhaibani

**Affiliations:** 1 Computer Center, University of Anbar, Ramadi 31001, Iraq; 2 College of Computer Science and Information Technology, University of Anbar, Ramadi 31001, Iraq; ahmed.abdalkafor@uoanbar.edu.iq (A.S.A.);; 3 School of Computer Science and Mathematics, Liverpool John Moores University, Liverpool L3 5AH, UK

**Keywords:** IoT-enabled supply chain, transportation and logistics scheduling, hybrid optimization algorithm, prism refraction search (PRS), operational efficiency

## Abstract

IoT-integrated supply chains play an important role in managing the movement of products and distribution, which relies on the processing of real-time data gathered using sensors and IoT-connected vehicles to make informed decisions that reduce logistical expenses. However, the optimization of transportation and logistics scheduling is still one of the most difficult tasks, which requires balancing demand and vehicle capacity, as well as delivery time in varying circumstances. This research assesses the performance capabilities and utility of four optimization algorithms, differential evolution (DE), a genetic algorithm (GA), simulated annealing (SA), and prism refraction search (PRS), which are applicable in IoT-integrated logistical processes. Notably, on the basis of the unique characteristics possessed by the four algorithms, a combination approach referred to as Bidirectional PRS-SA Optimization (Bi-PRS-SA) was formulated. This method ideally exploits the strengths of global and local searches within the search space. Furthermore, the research aims to discuss the proposed conceptual framework for integrating the proposed strategy into an overall IoT framework that would initiate dynamic supply chain management through the adaptation of the proposed strategy. Results show that the proposed strategy is better than the existing strategies of DE, GAs, SA, and PRS in terms of an overall range of 15–25%. Statistical validation via the Wilcoxon signed-rank test confirms these improvements are significant (*p* < 0.05). The findings suggest that the Bi-PRS-SA framework offers a robust and scalable solution for real-time logistics management in IoT-enabled environments.

## 1. Introduction

Supply chain systems create a significant framework for the development and functioning of today’s economic dynamics toward effectively and directly managing production systems, as well as information flow all the way along the path from the manufactures up to the customers. The main goal of most supply chain systems is to effectively address varying demands while, at the same time, striving to optimize their operational costs and make their systems economically sustainable [1]. The prevailing technology trends have increased the above aspects using Internet of Things-based applications to ensure the constant collection of data, utilizing connectivity and logistics systems carried on vehicles, etc.

One of the most important segments of the overall supply chain management function, transportation and distribution activities, is at the same time perhaps among the most difficult to effectively manage. The optimization of transport and logistics schedules involves satisfying multiple constraints, such as vehicle capacity and delivery times, for route optimization. This problem type is classified as NP-hard, implying that as problem complexity and scale grow, so does the difficulty of identifying an optimum solution [2,3]. The added complexity of real-time information in an IoT environment further adds to this problem complexity.

Supply chain management involves a group of integrated activities for coordination and efficient production, transport, and supply of goods and services [4,5]. The increasing rate of global trade and level of interconnectedness in the market necessitate highly efficient supply chains to maintain a competitive advantage for a particular company. This is worsened by the increased complexity and variety of disruptions that, if not well addressed, lead to lower degrees of efficiency [6,7]. In this regard, Internet of Things (IoT) technology improves visibility within the supply chain; however, for its efficiency, better forms of optimization are required.

In this regard, the overall effectiveness of transport and distribution activities is greatly dependent on the decision-making process. Based on [8], these decisions pertain to supplier selection, production planning, route determination, and inventory control, among others, which are strongly linked with each other. In IoT-enabled supply chains, enhanced data availability creates new opportunities for optimization while boosting computational burden due to real-time processing and usage of gained information.

Because of the complexity involved in scheduling in the domains of transport and logistics, metaheuristics have emerged as a popular paradigm. Metaheuristics are highly suitable for use in the handling of large optimization problems with a vast number of constraints assumed in optimization models. Additionally, metaheuristics possess the adaptability to be able to provide quality solutions in a reasonable computation time [9,10,11]. Metaheuristics have also been shown to be remarkably effective in solving real-case industrial optimizations, in which concise models are not a possibility [12]. Current research also suggests that hybrid metaheuristics tend to perform better than solitary metaheuristics in solving a given optimization problem concerning their quality of performance [13].

With these aspects in mind, the proposed work in this research seeks to improve the efficiency of transport and logistics planning in the IoT-based supply chain. The proposed work begins with an evaluation and comparison of the performance of four already established optimization algorithms, namely differential evolution, genetic algorithms, simulated annealing, and the prism refraction search, that help to deal with transport and logistics. Based on the potential already identified in the above approaches, a hybrid optimization strategy is proposed.

The design will facilitate intelligent transport logistics scheduling by reducing the transportation cost while increasing the efficiency of routes, along with the stability of solutions in relation to increased scales and complexity. Through the consideration of the functional requirements for hybrid methodologies in data-intensive IoT supply chain scenarios, this research makes contributions to the advancement of adaptive decision-support systems for supply chain logistics. The specific contributions of the current research are three-fold:

1. A systematic comparative analysis of four foundational metaheuristics—differential evolution (DE), genetic algorithms (GAs), simulated annealing (SA), and prism refraction search (PRS)—is established within the context of Capacitated Vehicle Routing Problems (CVRP). The assessment will extend beyond the traditional scope to provide a rigorous assessment of the underlying trade-offs between computation and quality in a variety of contexts.
2. This paper introduces the Bi-PRS-SA algorithm, which can be differentiated based on the concept of the bidirectional integration between PRS and SA. Rather than following the traditional trend and concept of the hybridization mechanism between the two optimization techniques, the proposed mechanism engages the SA technique for the optimization and improvement of the performance and convergence stability of the elite and low-performing solutions.
3. The proposed approach is subjected to an exhaustive experimental analysis in terms of a multidimensional set of performance measures. The investigation of best-, average-, and worst-case scenarios for the proposed approach allows for a statistically justified validation of reliability and the potential for coping with stochastic features of supply chain management.

The rest of the paper is organized as follows: Section 2 provides the related works. Section 3 outlines the methodology, starting with the mathematical formulation in Section 3.1 (detailing the objective function and constraints). Section 3.2 describes the proposed algorithms. Section 3.3 describes the theoretical analysis. Solution representation is discussed in Section 3.4, and Section 3.5 discussed IoT data integration architecture. Section 4 presents the results and discussion, covering the dataset, experimental setup, and findings. Finally, Section 5 concludes the paper.

## 2. Related Work

The rise in the usage of the IoT-enabled supply chain environment has caused transportation and logistics scheduling to emerge as one of the key considerations in minimizing costs and optimizing the efficiency of the supply chain. In recent years, the emergence of advanced studies has been increasing significantly, with the development of models that are able to optimize conflicting parameters such as the maximum carrying capacity of the vehicle, the across-the-board availability constraint, and the green constraint [14,15].

The study [8] investigated the use of a genetic algorithm (GA) for solving the Vehicle Routing Problem with Cross-Docking and Multiple Shipping (VRPCD&MS). The proposed GA-based solutions achieved an optimality gap of less than 5% when compared with branch-and-bound methods, indicating strong robustness and competitiveness. In the context of grain distribution systems in India, research in [16] employed both standard GAs and quantum-inspired GAs (Q-GAs) to optimize vehicle allocation decisions. Although the accuracy achieved by LINGO 18 was slightly higher, the computation time taken by the approaches based on GA was significantly shorter, and the reduction in transportation cost and CO2 emissions was equally significant. In a related study [17], the optimization of the Egyptian vegetable transport chain by combining binary particle swarm optimization for facility locations and ant colony optimization for routing was able to lower transportation wastage by 31% and enhance accessibility for small-scale farmers to the market. Handling uncertainties associated with the demand for transport services, a study carried out by [18] merged the whale optimization algorithm and the league championship algorithm using a fuzzy programming approach that increased transport profits by 22% by adjusting prices and levels of inventory accordingly.

In the study [19], a hybrid MPGA-VNS was created to address the logistical context of perishable products, taking into account the limitations of shelf life and the effects of temperature-sensitive routing. The experiment resulted in the achievement of a 17% cost reduction with respect to spoilages, which emphasizes the effectiveness of the integration of product decay with the routing framework. In the context of carbon cap-and-trade policies, in study [20], the MINLP approach was implemented for the problem. In this problem, the H-2 hybrid method resulted in the achievement of a 15% reduction of carbon with the optimization of the routing problem with respect to vehicle capacity and fuel indices on a real-time basis. In the context of the sugarcane industry, the study presented by the authors of the paper published in [21] resulted in the achievement of cost savings of 8.3% due to the optimization of the utilization of by-products with the implementation of the H-KASA method.

The problem of dynamic routing challenges was addressed by the authors of the paper published in [22]. In this work, the authors proposed an adaptive ant colony optimization algorithm. A scheduling accuracy of 99.9% was achieved with the proposed algorithm. In addition to this, the authors were also able to obtain cost savings of 14%. In the field of semiconductors, the authors of the work published in [23] addressed the problem of logistics scheduling using a two-layered network flow approach. Stochastic batch processes were optimized using techniques for computing the maximum flows. This approach improved storage utilization by 10% and enhanced scheduling accuracy by 13%. The work [24] explored warehouse kitting optimization by integrating ACO with GAs, resulting in a 24% reduction in energy consumption through a more balanced exploration–exploitation process. Finally, research in [25] addressed large-scale traveling salesman problems (TSPs) by introducing coevolutionary chains that accelerated convergence and improved path accuracy by 22% via automated dimension reduction techniques. Beyond routing-focused formulations, scheduling problems in distributed and IoT-enabled environments have also received significant attention, particularly through intelligent and secure workflow scheduling approaches that support data-driven decision making [26]. A summary of related work in logistics and transportation optimization is provided in Table 1.

Notwithstanding the foregoing improvements, there are obvious limitations of logistics scheduling solutions utilizing metaheuristics, brought to attention by the above-referenced literature. Scalability is still a significant issue, as solutions to date have difficulty in handling large-scale topological situations. Moreover, there are obvious limitations in handling either the accuracy of solutions or their time efficiency, coupled with a substantial need to address problematic parameter sensitivities. In addition to this, there is a significant lack of alignment with reality in addressing the contemporary needs of sustainability, to say the least. In this case, to fill the aforementioned limitations, a new hybrid optimization solution is proposed in this work, known as the Bi-PRS-SA method; this method will help in addressing the challenges of dynamic exploration and search intensification in logistics environments.

## 3. Methodology

The methodological approach used for this paper is based on a systematic performance evaluation of four prominent optimization methods, namely, differential evolution (DE), genetic algorithms (GAs), simulated annealing (SA), and prism refraction search (PRS), aimed at designing a hybrid optimization solution. In the context of IoT-realized logistics, where data is constantly being created as a result of IoT connectivity among transportation systems, the proposed solution tends to optimize transportation and logistics, thus finding a close-to-best solution to a rather complicated scheduling problem.

**Figure 1:**
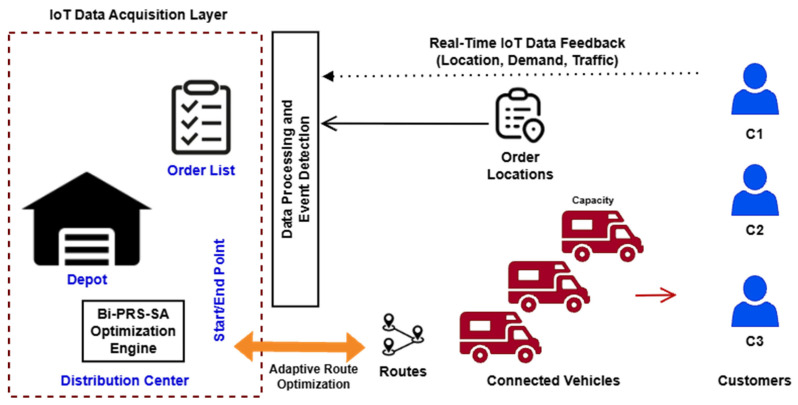
Conceptual architecture of IoT-enabled transportation and logistics scheduling, illustrating real-time data acquisition from connected vehicles and customers, data processing and event detection, and adaptive route optimization using the proposed Bi-PRS-SA algorithm.

The key to the methodology regards the reduction in the operational time necessary to obtain the solution, while keeping the solution stable by integrating global searching ability and a local optimization technique. The proposed algorithm has the potential to provide effective decision-making support concerning dynamic logistic tasks without causing additional computational burden by utilizing the strength of the chosen algorithms. Figure 1 describes a typical example related to the distribution of the logistics in the supply chain.

This paper is founded on a number of basic premises, which are significant to the application of the study:

1. Accurate data on demand, locations, and costs are available.
2. Locations are defined as geographic points.
3. No unexpected changes occur during implementation.
4. Vehicles have identical capacities and start/end at a fixed depot.
5. Roads are always accessible, with no traffic considerations.
6. Each client is served by one vehicle, respecting capacity limits.

In IoT-enabled supply chains, these assumptions provide a controlled baseline for evaluating optimization performance before incorporating real-time data variability. Note that the above assumptions are considerably idealized compared to real-world logistics, despite making the model formulation simpler. For instance, there may be errors and/or time delays within the data of the demand and the costs. Additionally, the depots’ and customers’ positions need not necessarily correspond to points, whereas the vehicles may carry different capacities and may face unforeseen stops such as traffic congestion. As a result, solutions derived under these perfect-information premises may overestimate achievable performance. Future work should thus focus on: integrating demand-forecast uncertainty via stochastic or robust optimization, relaxing the fixed depot and identical fleet assumptions by allowing heterogeneous vehicle profiles, and embedding real-time traffic data streams to adapt routes dynamically. These extensions will bridge the gap between our theoretical framework and operational deployment.

### 3.1. Mathematical Formulation

This work considers a vehicle routing and transportation scheduling problem under a single-depot routing structure with a homogeneous vehicle fleet. Each customer is associated with a deterministic demand and must be served exactly once by a single vehicle. All vehicles start and end their routes at the depot. The objective is to minimize the total traveled distance under vehicle capacity constraints.

#### 3.1.1. Sets and Indices

To formally define the routing structure and decision space of the problem, the following sets and indices are introduced:

- n: number of nodes in a route.
- p_0_, p_1_, p_2_, …, p_n_: set of all nodes, where node p_0_ represents the depot and nodes p_1_ to p_n_ represent customers.
- R = {r_1_, r_2_, …, r_|R|_}: set of vehicle routes.
- r ∈ R: index of routes.

#### 3.1.2. Parameters

The parameters used in the mathematical formulation are defined below:

- (x_i_, y_i_): cartesian coordinates of node *i*.
- d(i,j): Euclidean distance between nodes *i* and *j*.
- qᵢ: demand of customer *i* (with q_0_ = 0 for the depot).
- Q: capacity of each vehicle (identical for all vehicles).
- C_Route_: total cost of a single route.
- C_Total_: total cost of all routes.

The distance between any two nodes *i* and *j* is computed using the standard Euclidean metric as follows, Equation (1):

(1) $$d\left(i,j\right)=\sqrt{{\left(x_{i}-x_{j}\right)}^{2}+{\left(y_{i}-y_{j}\right)}^{2}}$$

where (*x_i_*, *y_i_*) and (*x_j_*, *y_j_*) denote the Cartesian coordinates of nodes *i* and *j*, respectively.

#### 3.1.3. Decision Variables

The decision variables describe the structure of each candidate solution in terms of vehicle routes and their associated service loads. Each solution is represented as a set of ordered routes, where every route specifies the visiting sequence of customers starting and ending at the depot. The total demand served along each route is implicitly determined by the customers assigned to that route and is used to verify feasibility with respect to vehicle capacity constraints.

#### 3.1.4. Objective Function

The main objective of this work is to determine a set of vehicle routes such that each customer is visited exactly once, vehicle capacity constraints are respected, and the total cost of all routes is minimized.

The cost of a single route is defined in Equation (2):

(2) $$C_{Route}=\sum_{i=1}^{n-1}d(p_{i},p_{i+1})+d(p_{n},p_{0})$$

where *C_Route_* is total cost of a route, *n* is number of nodes, and *p_i_* is the *i*-*th* node in the route.

The total cost of all routes is defined as in Equation (3):

(3) $$C_{Total}=\sum_{r\in R}C_{Route}$$

#### 3.1.5. Constraints

The most important restrictions that must be adhered to are:

1. Customer Coverage

Each customer must appear in exactly one route:

$$C={⋃}_{r\in R}r\;\mathrm{and}\;r_{a}\cap r_{b}=\;\emptyset \;\;\;\;\;\forall \;r_{a}\neq r_{b}$$

where $r_{a}$ and $r_{b}$ represent two distinct routes from the set of routes R.

This constraint ensures that each customer is visited exactly once and assigned to a single route.

2. Depot Constraint

Each route starts and ends at the depot:

p_0_ = 0, pₙ = 0

3. Capacity Constraint

For a decoded vehicle route *r* containing a set of customers, the total route load is defined in Equation (4):

(4) Load(r)=∑qᵢ 

where qᵢ is demand associated with customer *i*.

The feasibility condition requires:

Load(r) ≤ Q

In order to allow for a rigorous performance assessment, the current formulation incorporates the assumptions related to the existence of a single distribution center and the homogeneity of vehicle specifications, directly mapping the parameters from the benchmark instances considered in the current study. Even though the formulation becomes less general, the assumptions allow for a reproducible analysis for the assessment of the optimization performance.

### 3.2. Proposed Algorithms

In this regard, four distinct algorithms, differential evolution (DE), a genetic algorithm (GA), simulated annealing (SA), and prism refraction search (PRS), are considered for implementation in the current work, and their efficiency and robustness in finding optimal solutions have been considered. A brief overview of these algorithms is provided below.

#### 3.2.1. Differential Evolution (DE)

The algorithm uses the differential evolution (DE) approach as its methodology. This algorithm is known for its capability to explore the solution space widely and efficiently because of a variety of reasons. First, the approach uses a variety of solutions to begin with and this helps in covering the solution space widely. Next, this approach uses mutation and crossover operations to explore new spaces in the solution space. Finally, this approach always selects the best solutions and this helps in converging into the optimal solution. The total cost is minimized by optimizing the distribution of requests among vehicles using parameters such as F and CR to adjust the variability of solutions. Algorithm 1 describes the DE algorithm.
**Algorithm 1:** Differential Evolution (DE)1.Initialization: Create an initial population of NP individuals (vectors) randomly within the search space.2.Evaluate Initial Population: Compute the fitness of each individual in the population and identify the best individual.3.Main Loop:Mutation: For each individual *x_i_* in the current generation, create a mutant vector *v_i_*, by Equation (5):                                         $v_{i}=\;x_{r1}+\;F\;*\;\left(x_{r2}-\;x_{r3}\right)$                                   (5)                                         where *x_r_*_1_, *x_r_*_2_, and *x_r_*_3_ are three randomly chosen individuals from the population (distinct from *x_i_*). *F* is a scaling factor (a real number, typically between 0 and 1) that controls the amplification of the differential variation.b.Crossover (Recombination): Create a trial vector *u_i_* by combining elements from the mutant vector *v_i_* and the target vector *x_i_*. A common crossover strategy is binomial or uniform crossover, as:                                         $\{\begin{array}{c} u_{ij}=\;v_{ij}if\;rand\left(0,1\right)<CR\;or\;j=j_{rand}\\ u_{ij}=x_{ij}\;\;\;otherwse \end{array}$                                         where CR is the crossover probability (a real number between 0 and 1). *j_rand_* is a randomly chosen index (1 to dimension of vector).c.Selection: Compare the trial vector *u_i_* with the target vector *x_i_*. If the trial vector has a better fitness value (according to the objective function), it replaces the target vector in the next generation.                                         $\{\begin{array}{c} x_{t+1}=\;u_{i}\;\;\;if\;f\left(u_{i}\right)<f(x_{i})\;//\mathrm{Minimization}\;\mathrm{problem}\\ x_{t+1}=x_{i}\;\;\;\;\;\;\;\;\;\;\;\;\;\;\;\;\;\;\;\;otherwse \end{array}$4.Stopping Condition: Repeat until a termination criterion is met.5.Output: Return the best solution.

#### 3.2.2. Genetic Algorithm (GA)

A genetic algorithm is a method of optimization and search. This algorithm (Algorithm 2) relies on a set of possible solutions to a problem, called a ‘population’. These solutions are developed through selection, hybridization, and mutation, to arrive at the optimal or near-optimal solution. Genetic algorithms are characterized by their ability to deal with complex problems that do not have a clear analytical solution, and they are used in a variety of fields.
**Algorithm 2:** Genetic AlgorithmInitial Generation: Generate random initial solutions (randomly initialize the population).Solution Evaluation: Calculate the total cost of solutions based on the objective function.Selection: Use the best solutions to pass on to the next generation.Crossover: Merge solutions to form new solutions.Mutation: Very simple modification of solutions randomly to improve diversity.Replacement: Generate a new population.Iteration: Repeat steps (2) to (6) for a certain number of generations.

#### 3.2.3. Simulated Annealing (SA)

The simulated annealing (SA) algorithm initiates a feasible starting solution and enhances it iteratively by searching in the neighborhood solutions using mutation operator. The acceptance of new solutions depends on the difference in cost between the current solution and the previous one. Using the current temperature, lower-quality solutions can be accepted to prevent local minima and enable controlled search. The algorithm gradually decreases the temperature with emphasis on convergence towards an optimal or near-optimal solution. Algorithm 3 is SA.
**Algorithm 3:** Simulated Annealing (SA)Set the initial temperature *T*_0_, cooling rate *α*, and maximum number of iterations *max_ite*.Generate a random solution as initial solution.Iteration Loop; repeat until *max_ite* is reached or the temperature drops below a certain threshold:Derive a new solution by small-mutating the current solution.Calculate the cost of the new solution.Accept new solution if *C_new_* < *C_current_*.Otherwise, accept by acceptance probability:                                                 $P=e^{\frac{C_{current}-C_{new}}{T}}$Update the best solution.Update the current temperature:                                                        T←T×αT4.Return the best solution.

#### 3.2.4. Prism Refraction Search (PRS)

Prism refraction search (PRS) [27] is a physical optics-inspired, metaheuristic algorithm drawn from the phenomenon of refraction of light as it moves through a triangular prism. PRS is a single-solution optimization algorithm. PRS relies on solution updating by simulating the process of refraction, with refraction angles computed according to physical laws to enable efficient searching for the best solutions within the solution space. PRS provides improved global exploration using refraction-inspired diversity, adaptive trigonometric search modulation, and continuous-space flexibility. PRS’s qualities allow the navigation of intricate logistics solution spaces and the avoidance of premature convergence. The use of the PRS algorithm represents a new contribution to the field of logistics optimization, as this is the first application of this algorithm in this field. Algorithm 4 is PRS.
**Algorithm 4:** Prism Refraction Search (PRS)Initialization: The initial set *i*_0_, which is the solution as the angle of incidence, is constrained by the prism angle *A*_0_, using Equations (6) and (7):                             $i_{0}=LB+\left(UB-LB\right)\times U\left[0,90\right]$                                                                   (6)                             where *LB* and *UB* are the lower/upper bounds, and *U*[0,90] is a random value between 0 and 90 degrees. *A*_0_ starts above 15 degrees to avoid premature convergence [27].                                 $A_{0}=\max\left(LB\right)+\left(\min\left(UB\right)-\max\left(LB\right)\right)\times U\left[15,90\right]$                             (7)For iterations *t* = 1 to *MaxIters*:For solution *i* = 1 to *OneSolution*:
●For each dimension *j*:
○Compute the fitness (*δ_t_*) by the cost function.○Update the *BestScore* to the current fitness, if the current fitness is better.Calculate refractive index *μ_m_*: using Equation (8):                             $\mu_{t}=\frac{\sin\left(\frac{A_{t}+\delta_{t}}{2}\right)}{\sin\left(\frac{A_{t}}{2}\right)}$                                                                                       (8)For solution *i* = 1 to *OneSolution*:
●For each dimension *j*:
○Update the emergent angle *E_t_* using Equation (9):                         $E_{t}=\delta_{t}-i_{t}+A_{t}$                                                                                   (9)Define a random number *r*_1_ in the range [−1, 1].Update the incident angle *i_t_*_+1_ using Equation (10):                         $i_{t+1}^{j}=\;{sin}^{-1}\left(-sinE_{t}*\;cosA_{t}+r\;*\;sinA_{t}*\;\sqrt{\mu_{t}^{2}-{sin}^{2}*E_{t}}\right)$              (10)Update prism angle *A_t+1_*: using Equation (11):                             $A_{t+1}=A_{t}\times \exp\left(\frac{-\alpha \times t}{MaxIter}\right)$                                                                      (11)                             where *α* is decay rate.Update best solution and position based on the improvements observed during the current iteration.3.Return the best solution.

#### 3.2.5. Proposed Bidirectional PRS-SA Optimization (Bi-PRS-SA)

After implementing the four algorithms and analyzing and comparing their performance, the SA and PRS algorithms were chosen as the basis for developing the new hybrid algorithm proposed in this work, Algorithm 5. The hybrid algorithm integrates the local search of SA into PRS to improve solution quality and avoid premature convergence. The proposed hybridization within the algorithms provides a balance between exploration and exploitation of the solution space.
**Algorithm 5:** Bidirectional PRS-SA Optimization (Bi-PRS-SA)Initialization: Population size (*P*), maximum iterations (*Max_I*), trigonometric modulation (*A_t_*), initial temperature (*initialTemp*), cooling rate (*α*).Generate a population of solutions.Generate an initial PRS population.PRS Evolution
Calculate the cost for each solution and track the global best solution.Apply trigonometric mutation to update the population by the parameter *A_t_*.SA-Driven and Hybridization Strategy
Identify the worst and best solutions in the PRS population.Apply SA-driven algorithm to refine these solutions:
○Generate neighboring solutions.○Accept solutions using the following:                                   $Probability=\;\{\begin{array}{c} 1\;,\;\;\;\Delta Cost<0\\ \exp\left(-\frac{\Delta Cost}{temperatureSA}\right),\;otherwise \end{array}$Update *temperature_SA_* using the following:                             ${temperature}_{SA}\;*=\;\alpha$Population Integration
Convert SA-improved solutions back into PRS population.Replace the worst PRS solutions with improved SA solutions to enhance diversity and improve population quality.Repeat, until *Max_I* reached.Return the best solution found.

The main design goal is to achieve an effective balance between exploration (discovering new promising routing structures) and exploitation (refining existing routes toward near-optimality). PRS provides strong global exploration through refraction-inspired diversification, whereas SA offers efficient local intensification via neighborhood search and controlled acceptance of inferior moves. However, using either method in isolation is insufficient: PRS may identify promising regions without adequate local refinement, while SA may stagnate in local optima without global guidance. To address these limitations, Bi-PRS-SA adopts a bidirectional integration strategy in which SA is selectively applied to both elite and low-quality PRS solutions, enabling simultaneous intensification and diversification with limited computational overhead. The complementary roles of PRS and SA within the proposed bidirectional hybrid algorithm are summarized in Table 2.

In the proposed Bi-PRS-SA algorithm, a bidirectional encoding mechanism is employed to ensure compatibility between the continuous search space of PRS and the discrete route representation required by SA. This mechanism enables effective information exchange between global exploration and local exploitation phases within a unified optimization process.

During the PRS phase, the candidate solution is represented as a continuous-valued vector. In the continuous-valued vector representation, each component of the vector has a corresponding customer and a corresponding priority value. To convert the PRS solution into a discrete route layout that can be accepted by the SA search engine, the continuous-valued vector first needs to be reordered according to the customer visit priorities. By doing so, a customer visitation sequence can be derived. To derive the routes that can be followed by the vehicles, the list needs to be segmented. This can be performed by iteratively inserting customer orders.

During the application of the SA method to the decomposition of the problem into individual routes, the method acts locally. In this respect, the neighboring solutions are obtained by modifying the current solution, for example, by swapping or shifting a customer to another route, with the capacity constraints remaining valid. In the evaluation of the candidate solutions, the Metropolis criterion is used.

The next step is the inverse transformation, where the refined discrete route is re-encoded to a continuous form and integrated back into the PRS population. This inverse transformation assigns a continuous value to the customers according to the revised visiting order, thereby preserving the structural improvement achieved during the SA refinement phase.

Each candidate solution is evaluated based on the objective function. Equation (1) is used for the computation of inter-node distances, Equation (2) for the computation of route costs, and Equation (3) for the computation of the total cost *C_Total_*. During capacity-aware decoding, route loads are tracked using Equation (4) and any infeasible solution triggers a repair operator that reallocates overloaded customers before cost evaluation. In the SA phase, neighboring solutions are assessed using the same cost and feasibility criteria and only moves that satisfy the constraints and are accepted under the Metropolis rule are retained.

The collaboration between PRS and SA operates in three complementary directions:

1. Exploitation, where SA intensifies the search by refining high-quality (elite) PRS solutions;
2. Exploration, where SA perturbs low-quality PRS solutions using temperature-controlled randomness to escape stagnation;
3. Bidirectional encoding, which dynamically transforms solutions between continuous and discrete representations to maintain coherence between both phases.

The combination of PRS’s powerful global exploration and SA’s accurate refinement can ensure an optimal balance of global and local exploration and exploitation. Therefore, the Bi-PRS-SA achieves outstanding performance on transportation and logistics scheduling problem optimization.

### 3.3. Theoretical Analysis

Convergence Properties: Based on mild assumptions regarding the modulation parameter *A_t_* (specifically, that *A_t_* approaches 0 as iterations increase), the trigonometric mutation operator of PRS shows global exploration at first and gradual intensification later. This behavior ensures the probabilistic coverage of the search space. When coupled bidirectionally with SA’s Metropolis criterion, every PRS iteration either improves the current best solution or accepts a worse one with nonzero probability. Standard results for SA then guarantee that, as temperature decays sufficiently slowly (cooling rate close to 1), the hybrid will converge in probability to a global minimum. In practice, choosing cooling rate and *A_t_* type conditions provides asymptotic convergence, though at the cost of slower runtime.

Computational Complexity: Let *N* be population size, *I* the maximum number of PRS iterations, and *n* the number of customers.

1. PRS decoding and fitness evaluation: Each iteration costs *O*(*N* · *n log n*) (the *log n* from sorting during decoding plus *O*(*n*) distance sums).
2. Trigonometric mutation over *N* individuals costs *O*(*N*) per iteration.
3. SA local searches are applied to two individuals per PRS iteration; each neighbor generation and evaluation costs *O*(*n*), and if you run *S* SA steps per call, that is *O*(*S* . *n*).

Hence, the overall worst-case cost is

O(I [ N(nlogn+1)+S n ])  =  O(IN(nlogn)+I S n)

In real-world tests we set *S* ≪ *N*, so the dominant term remains O(I N n log n). This analysis shows that Bi-PRS-SA scales polynomially in problem size, with the major drivers being population size and decode cost.

### 3.4. Solution Representation

**Figure 2:**
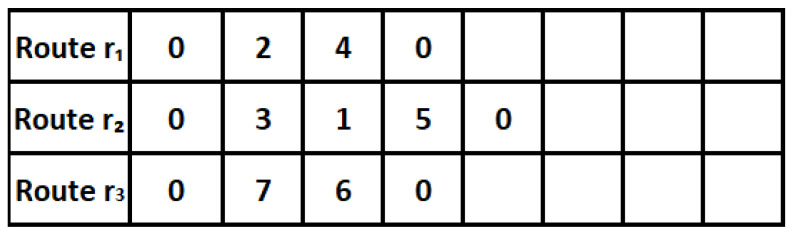
Route-based solution representation after decoding.

The solution representation should reflect the characteristics of the vehicle routing problem and describe the scheduling for each vehicle. The solution representation in the proposed model shows how to express the routes in the transportation and distribution process within supply chains. The representation is based on a design of a set of variables that define the vehicles used, the locations (nodes) visited, and the paths between the nodes. To represent the solution, a chromosome is defined in the form of a binary matrix of the number of customers multiplied by the number of vehicles. The proposed representation matrix enables us to choose any customer for any vehicle. Figure 2 illustrates an example of a solution representation. Here, the rows denote the vehicles, and the columns denote the position within the sequence of the customers. The numbers, like the 2 in the first row (R1), denote that the first vehicle goes to the second customer first, while the 4 in the first row denotes that the next visit in the sequence is to the fourth customer. The blank space denotes that the route has come to an end.

Mutation and crossover operations are applied to improve diversity and ensure efficient exploration. Mutation includes several strategies, such as swapping two cities within the same route, adding a new city to a given route, deleting an unnecessary city, creating a new route by redistributing cities from different routes, or merging two routes into one to improve efficiency. Crossover relies on various techniques, such as single-point intersections where parts are exchanged after a given point, two-point intersections where parts are exchanged between two points, regular intersections where random locations are chosen for part exchange, and finally partial intersections which merge a part from one of the parents into the new solution, enhancing the search for better solutions. It is important to ensure that the operations applied maintain the validity of the solution, i.e., they do not violate problem constraints such as vehicle capacity or visiting all cities. Method operations preserve solution feasibility through constraint-aware design. Crossover exchanges complete routes to avoid duplicate customer assignments. Modifications via mutation are only made to sequences after capacity limits have been verified. This ensures that all solutions remain valid throughout the optimization process.

### 3.5. IoT Data Integration Architecture

IoT-powered supply chains always have a continuous stream of data being produced, coming from a network of interconnected sensors. In these locations, there is a need for making decisions that can change or adapt in real time, unlike other vehicle routing problems. Therefore, to fill the gap that exists between optimization theory and IoT-powered logistics, there is a need to present a new concept of IoT’s integration with its respective data, which will be presented along with a new optimization algorithm, Bi-PRS-SA.

The architecture takes into account multiple types of IoT data sources commonly used in modern logistics systems, including the following:

1. GPS sensors mounted on vehicles for real-time location tracking.
2. Environmental sensors (e.g., temperature and humidity) for monitoring sensitive goods.
3. Traffic and road-condition data obtained from intelligent transportation systems.
4. Demand sensors or enterprise IoT platforms that continuously update customer requests.

Heterogeneous data streams are ingested through IoT gateways and channeled into a processing layer, which may be configured as either a centralized or edge-based architecture. Within this level, the raw telemetry information goes through a filtering and aggregation process to arrive at optimization parameters that can be acted on. For example, the dynamics in the demand placed on the load constrain the parameters, while the informatics on traffic flow affect the actual distance traveled. The ongoing vehicle telemetry also aids in the determination of any deviation in the routes taken or any delays that may have occurred. The move away from the traditional one-shot approach to decision-making involves the use of periodic or trigger-based events.

The Bi-PRS-SA algorithm essentially performs the roles of a computational core within this model. The system will initiate a partial re-optimization of an existing routing solution upon identifying large changes in system variables. The PRS component will aid in exploring and identifying alternative routing structures, and the SA component will assist in refining both high-tier and suboptimal solutions. This will ensure greater adaptability to changes in variables without re-executing the entire system.

It should be noted that this specific study makes use of controlled benchmark sets to ensure rigor for comparative purposes. However, the underlying architecture represents the potential for the scalability of the Bi-PRS-SA approach within the IoT environment. Real-time sensing, adaptive triggers, and hybrid decision-making represent a potential trajectory for the development and implementation of data-centric logistics systems. Future potential avenues of inquiry might include the potential for edge computing.

## 4. Results and Discussion

Three commonly referenced benchmark sets have been used to evaluate the performance of the algorithms, as described by the Augerat team [28]. Although the Augerat test problems are artificially created, they have a well-analyzed pattern of spatial distribution.

In order to test and measure the effectiveness of this proposed algorithm, this study utilizes three different sets of Augerat benchmark problems, namely Sets A, B, and P, each offering a variety of spatial and demand configurations.

1. Augerat Set A: This consists of 27 instances where nodes are randomly distributed within a Cartesian plane of dimensions 100 × 100. Each node is assigned an average demand of about 15 units. However, for 10% of these nodes, their demands are tripled. In addition, the capacity of the vehicles is held constant at 100 units.
2. Augerat Set B: This set comprises 23 instances with the feature of spatial clustering, wherein the number of clusters has been intentionally set greater than the available fleet size. Demand ranges between 1 and 30 units, with the same 10% high-demand outlier strategy used for the instances in Set A.
3. Augerat Set P: This set consists of modified versions of the traditional benchmark problems. These problems have the advantage that the performance can be compared against the traditional computational standards.

The choice of dataset allows for a controlled validation scenario in which the effects of a small-to-medium problem size, as well as a varied demand topology (both uniform and clustered), can be used to perform a detailed assessment of algorithm performance. The incorporation of hotspot nodes, comprising 10 percent of the locations, allows for a performance assessment of solution flexibility in a controlled environment.

In order to ensure fair and unbiased assessment for the different optimization methods being considered, all the methods are evaluated within the context of the exact same conditions. In this regard, the differential evolution method, genetic algorithm, simulated annealing method, prism refraction search method, and the proposed method are all implemented within the context of the exact same number of iterations. This ensures the assessment is fair and unbiased.

All algorithms have been run with an equivalent number of fitness function evaluations. For population-based approaches, for every iteration, all candidates are evaluated for DE, GA, PRS, and Bi-PRS-SA. The number of evaluations for SA has been adjusted to correspond to an average number per iteration, similar to population-based approaches. Thus, none of these optimization techniques have an unfair advantage by means of additional function evaluations. Furthermore, the experiments with each algorithm have been performed 30 times for each instance using different random seeds. This accounts for the stochastic nature of the algorithms. The experiments have been performed on a consistent hardware and software configuration. In addition, the stopping criteria have been the same for all the algorithms. Hence, the experiments have been performed with a fair comparison among all the algorithms. In the experiments, all the algorithms have been implemented using the Java programming language. The parameter settings for the algorithms are presented in Table 3, Table 4, Table 5, Table 6 and Table 7.

The efficiency of the algorithm was checked by computing the average of the total distance of the generated routes for 30 executions. Differences between the distributions of the proposed Bi-PRS-SA algorithm and other algorithms are shown using box–whisker plots. In the experiments, four algorithms are compared—DE only, GA only, SA only, and PRS only—with the same settings. In addition, the Bi-PRS-SA algorithm was included. All the algorithms were executed with the same settings, and the obtained values were registered to obtain the average cost. Box plots show the distribution of the obtained results and the efficiency of the approach.

To give a comprehensive assessment of the performance of the algorithm, as well as the mean route distance, several other statistical measures were used. For every benchmark problem, and for every algorithm, the following measures were calculated for 30 different runs:

- Best Cost: The smallest cost for the routes.
- Worst Cost: The maximum total route distance obtained.
- Mean Cost: The average route distance, used for general performance comparison.

Moreover, the nature of the convergence behavior was analyzed by tracking the value of the best known solution that has been obtained up to a certain number of iterations. From the plots showing the convergence curves, it can be observed that the rate at which the algorithms reach high-quality solutions varies. This analysis confirms that the presented Bi-PRS-SA not only finds better-quality solutions but also converges faster than the other algorithms. The mean, best, and worst solution costs for all three groups are presented in Table 8.

**Figure 3:**
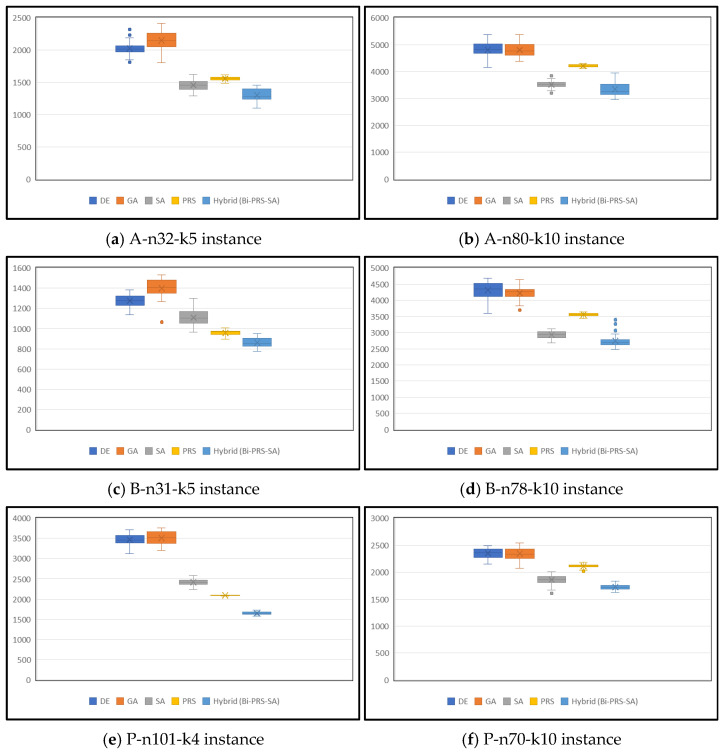
Box-and-whisker plots showing the comparison of Bi-PRS-SA with other algorithms.

The box-and-whisker plots used in Figure 3 are for comparing Bi-PRS-SA with the baseline algorithms. The box-and-whisker plots effectively show the stability of our proposed combination.

The hybrid model for which the proposed hybrid algorithm has been developed combines global search power through its integration with PRS and local search power through its integration with SA. The hybrid model provides a complete search technique. The adaptive diversity technique helps avoid premature stagnation, and a better approach for increasing computational efficiency involves applying SA only for the best and worst candidates. In IoT-based supply chain scenarios where routing decisions have to be made in data-intensive and highly dynamic scenarios, the proposed technique provides a well-rounded approach with which an optimal balance between efficiency and quality has been achieved.

The robustness and dependability of the performance of Bi-PRS-SA were also validated visually using box-and-whisker plots and statistically using the Wilcoxon signed-rank test with a significance of *p* < 0.05. From the findings, it can be concluded that the performance gains achieved are consistent and are not attributed to any randomness.

Though the experimental study performed on the artificial benchmark problems provides a clear demonstration of the major advantages that the Bi-PRS-SA approach offers, the logistics problems that exist in the real world have other issues that must be taken into account. For real-world supply chains (especially those enabled by the presence of IoT infrastructure), there are stochastic demand behaviors, hard time constraints, and diverse vehicle capacities that are not reflected well by the Augerat instances. For these reasons, future studies should shift their attention towards the development of models that would consider time-varying traffic velocities, real-time demand forecasting models, and self-regulating parameter calibration systems that can adjust according to the dynamically changing environment of the logistics problem.

## 5. Conclusions

In this work, the importance of using different optimization algorithms has been demonstrated to enhance the efficiency of transport and logistics routing in the supply chain system. The experimental outcome shows the proposed approach named Bi-PRS-SA does better than the other algorithms used for the comparison task. This will ensure the attainment of a well-balanced outcome in terms of execution speed. This will prove the viability of the integration of global search and different techniques of local search to obtain better results. This integration will utilize different algorithms to come up with better solutions to logistical issues.

Moreover, the achieved results substantiate that the proposed optimization approach acts as a reliable set of tools that can be used to make the necessary decisions in logistics scheduling problems characterized by high complexity. With respect to the application of the proposed methodology in the context of Internet of Things-based SCs, it must be noted that the flexibility of the SCs relies on the permanent provision of telemetry information regarding the interconnected assets.

Even though these metrics show promising results, the scope of this research follows specific boundaries based on methodology. The validation was based on standardized benchmarking data. Though deterministic and precise, this does not completely account for random variations that may occur in an actual IoT network. Additionally, this model only allows for a single depot and a homogeneous fleet. Although this alignment was necessary to maintain experimental parity with established benchmarks, it may not reflect the multifaceted requirements of diverse, real-world industrial deployments.

In terms of future work, it is proposed that further research be conducted to incorporate a wider range of practical and varied logistics scenarios, such as diverse vehicle fleets and information that varies over time, such as traffic conditions. Moreover, it has been noted that data-driven approaches to parameter control, as well as extension of the proposed optimization problem to consider multiple objective functions, are promising avenues of future work. The former would likely aid in improving the applicability of the proposed method to Internet of Things-enabled supply chains.

## Figures and Tables

**Table 1:** Summary of related work in logistics and transportation optimization.

Ref.	Problem/Context	Method	Efficiency/Cost	Sustainability	Key Results
[8]	Vehicle Routing with Cross-Docking and Multiple Shipments (VRPCD&MS)	Genetic algorithm (GA)	Achieved < 5% optimality gap compared to branch-and-bound with reduced computation time	-	GA-based solutions demonstrated strong robustness and competitiveness
[16]	Sustainable agro-food grain supply chain (India)	GA and quantum-inspired GA (Q-GA)	Significant reduction in transportation cost with faster computation than exact solvers	Reduced CO_2_ emissions	GA-based methods achieved near-optimal solutions with lower runtime
[18]	Location–inventory–pricing in closed-loop supply chains under uncertainty	Hybrid whale optimization + league championship algorithm with fuzzy programming	Increased transport profit by 22%	Sustainability considered via closed-loop modeling	Demonstrated robustness under demand uncertainty
[19]	Perishable product logistics with shelf-life and temperature constraints	Hybrid MPGA–VNS	Reduced spoilage-related costs by 17%	Indirect sustainability via waste reduction	Highlighted effectiveness of hybrid metaheuristics for perishables
[20]	Supply chain optimization under carbon cap-and-trade policies	MINLP + H-2 hybrid metaheuristic	Optimized routing and vehicle usage in real time	Reduced carbon emissions by 15%	Demonstrated trade-off between cost and emission reduction
[21]	Multi-echelon sugarcane closed-loop supply chain	H-KASA hybrid metaheuristic	Improved logistics efficiency by 8.3%	Addressed sustainability via by-product reuse	Showed benefits of hybrid methods in agro-industrial networks
[22]	Dynamic logistics scheduling under volatile conditions	Adaptive ant colony optimization	Reduced operational costs by 14%	-	Achieved scheduling accuracy of 99.9% in dynamic environments
[23]	Semiconductor logistics scheduling	Dual-layer network flow optimization	Improved storage utilization by 10%	-	Enhanced scheduling accuracy by 13%
[24]	Warehouse kitting in green supply chains	Hybrid ACO–GA	Improved operational efficiency	Reduced energy consumption by 24%	Demonstrated benefits of balanced exploration–exploitation
[25]	Large-scale traveling salesman problem (TSP)	Coevolutionary-chain-based ACO	Accelerated convergence and improved accuracy by 22%	-	Effective dimension reduction for large-scale routing

Note: (-) denotes not considered or not reported in the study.

**Table 2:** Complementary roles of PRS and SA in the proposed Bi-PRS-SA algorithm.

Component	Strength	Weakness If Used Alone	Role in Bi-PRS-SA	Why Bidirectional Is Beneficial
PRS (global search)	Strong exploration; refraction-based diversification helps escape premature convergence	May produce good global regions but less refined local solutions (no strong intensification)	Generates diverse candidate routes and explores search space widely	Provides high-quality and diverse solutions that SA can exploit/refine
SA (local search)	Strong exploitation; neighborhood-based refinement improves route quality quickly	Can become trapped in local minima if not guided by global exploration	Refines solutions via controlled acceptance (Metropolis) to improve feasibility/cost	When applied to selected PRS solutions, it improves both convergence speed and stability
SA on elite PRS solutions	Intensifies search around promising basins	Risk of losing diversity if used excessively	Improves best candidates (exploitation)	Raises final solution quality without restarting PRS
SA on poor PRS solutions	Controlled perturbation introduces structured diversity	Randomness alone may be inefficient	Diversifies poor candidates (exploration)	Prevents stagnation and injects new regions into PRS population
Integration back to PRS population	Maintains a population-level learning dynamic	Without reintegration, gains remain local	Replaces worst PRS solutions with SA-improved ones	Creates a feedback loop that balances exploration/exploitation more effectively than one-way hybrids

**Table 3:** DE algorithm parameter values.

Parameters	Value
Scaling factor (F)	0.8
Crossover rate (CR)	0.7
Population size	100
Number of generations	1000

**Table 4:** GA parameter values.

Parameters	Value
Population size	100
Number of generations	1000
Crossover rate	0.8
Crossover type	Two-point
Mutation rate	0.05
Selection	Truncation selection

**Table 5:** SA algorithm parameter values.

Parameter	Value
Initial temperature	1000
Cooling rate	0.995
Max iterations	1000

**Table 6:** PRS algorithm parameter values.

Parameters	Value
Number of solutions in each generation	100
Max iterations	1000
Alpha	0.09

**Table 7:** Bidirectional PRS-SA Optimization (Bi-PRS-SA) algorithm parameter values.

Parameters	Value
Number of solutions in each generation	100
Max iterations	1000
Alpha	0.09
Initial temperature	1000
Cooling rate	0.995

**Table 8:** Results (mean, best, and worst) of algorithms.

Instance	DE			GA			SA			PRS			Hybrid (Bi-PRS-SA)		
	Mean	Best	Worst	Mean	Best	Worst	Mean	Best	Worst	Mean	Best	Worst	Mean	Best	Worst
A-n32-k5	2027.8	1810	2322	2150.7	1805	2415	1456.9	1289	1624	1557.7	1484	1617	1300.6	1100	1454
A-n33-k6	1723.3	1592	1946	1693.4	1487	1927	1306.9	1160	1449	1207.7	1153	1250	1102.1	966	1190
A-n36-k5	2093.7	1918	2294	2089	1825	2323	1421.1	1257	1557	1525.5	1429	1588	1281.1	1185	1452
A-n44-k6	2386.7	2108	2674	2347.6	2007	2689	1674.4	1484	1892	1946.6	1877	2017	1586.4	1466	1755
A-n45-k7	2630.1	2263	2882	2617.8	2315	2909	1878.9	1767	2037	2068.3	1924	2124	1769.7	1630	1990
A-n53-k7	2907.4	2310	3175	2924.5	2574	3197	2076.4	1883	2301	2452.6	2306	2527	1932.7	1754	2143
A-n60-k9	3610.7	3267	3997	3583	3308	3925	2560.1	2346	2847	2988	2911	3055	2504.8	2230	2825
A-n62-k8	3777	3295	4174	3755.5	3149	4054	2633.7	2336	2871	3018	2859	3091	2453.7	2202	2830
A-n63-k10	3393.3	2937	3790	3456.3	3132	3752	2597.2	2377	2905	2937.9	2809	3004	2454.4	2222	2801
A-n80-k10	4811	4150	5376	4806.3	4390	5385	3511.8	3199	3842	4213.8	4131	4300	3349.1	2958	3948
B-n31-k5	1275.1	1139	1385	1399.7	1065	1531	1110	963	1300	958.5	897	1006	859.8	775	951
B-n35-k5	2544.5	2142	2901	2582.8	2149	2994	1596	1436	1764	1801.9	1678	1879	1504.7	1316	1749
B-n39-k5	2272.2	1920	2704	2210.8	1724	2849	1322.7	1162	1446	1378	1253	1469	1147.4	954	1300
B-n41-k6	2491	2097	2841	2390	2066	2876	1580.6	1459	1843	1701.1	1593	1799	1447.6	1333	1604
B-n44-k7	2414	2113	2671	2391.4	1898	2737	1685.6	1530	1927	1840.9	1740	1903	1543.8	1387	1733
B-n50-k7	2740.7	2449	3085	2699.6	2270	3037	1840.2	1617	2129	2061.2	1968	2165	1713	1453	1978
B-n63-k10	4159.5	3522	4590	4076.1	3682	4558	2931.9	2709	3215	3335	3157	3449	2750	2551	3230
B-n66-k9	3578	3245	3946	3641.6	3133	3915	2491.4	2279	2758	2947.8	2836	3027	2286.3	2086	2642
B-n68-k9	3960.7	3561	4391	3962	3399	4417	2624.7	2343	2951	3219.7	3088	3300	2416	2189	2887
B-n78-k10	4318.2	3590	4682	4218.8	3694	4638	2934.3	2681	3115	3558.6	3434	3635	2743.5	2475	3399
P-n101-k4	3472	3117	3716	3512.1	3207	3760	2417.5	2243	2592	2091.4	2076	2104	1648.5	1571	1727
P-n16-k8	512.9	436	589	502	403	626	490.6	431	559	458	450	464	455.7	450	462
P-n19-k2	360.1	317	377	477.3	407	567	316.3	277	361	311.7	294	329	267.4	234	298
P-n20-k2	380.6	354	405	474.4	410	571	321.4	283	359	325.1	302	339	275.2	240	316
P-n22-k2	410.9	358	435	515.9	434	601	347.7	305	397	357	335	374	280.2	226	323
P-n40-k5	1304.3	1212	1448	1310.5	1186	1440	978.3	852	1084	949.7	917	972	784.1	696	862
P-n50-k10	1595.8	1406	1766	1589.1	1419	1799	1331.5	1232	1423	1369.7	1291	1401	1219.1	1131	1320
P-n50-k7	1648.7	1443	1848	1666.1	1518	1832	1236	1144	1412	1198.5	1165	1221	1058.6	956	1129
P-n60-k10	1975.2	1802	2218	1973.1	1772	2141	1617.5	1512	1806	1670.4	1610	1722	1440.5	1318	1532
P-n70-k10	2348.3	2147	2492	2343.9	2069	2541	1861.7	1611	2007	2109.8	2019	2186	1726.8	1626	1838

## Data Availability

The data used in this study are available from publicly accessible sources.

## References

[1] Christopher M. (2016). Logistics and Supply Chain Management: Logistics & Supply Chain Management.

[2] Emenike S.N., Falcone G. (2020). A review on energy supply chain resilience through optimization. Renew. Sustain. Energy Rev..

[3] Fathollahi-Fard A.M., Dulebenets M.A., Hajiaghaei–Keshteli M., Tavakkoli-Moghaddam R., Safaeian M., Mirzahosseinian H. (2021). Two hybrid meta-heuristic algorithms for a dual-channel closed-loop supply chain network design problem in the tire industry under uncertainty. Adv. Eng. Inform..

[4] Kavilal E.G., Venkatesan S.P., Kumar K.H. (2017). An integrated fuzzy approach for prioritizing supply chain complexity drivers of an Indian mining equipment manufacturer. Resour. Policy.

[5] Awad H., Aboalganam K., Hijazeen O., Alhanatleh H., Moh’d Abu Bakir S., Altahrawi M., Kilani Y.M., Malahmeh H.M. (2023). Investigation study of the cloud supply chain management system. Ing. Des Syst. D’inf..

[6] Chowdhury N.A., Ali S.M., Mahtab Z., Rahman T., Kabir G., Paul S.K. (2019). A structural model for investigating the driving and dependence power of supply chain risks in the readymade garment industry. J. Retail. Consum. Serv..

[7] Piya S., Shamsuzzoha A., Khadem M. (2020). An approach for analysing supply chain complexity drivers through interpretive structural modelling. Int. J. Logist. Res. Appl..

[8] Gnanapragasam S.R., Daundasekera W.B. (2024). Meta-heuristic method to schedule vehicle routing with moving shipments at the cross-docking facility. Natl. Sci. Found..

[9] Abualigah L., Hanandeh E.S., Zitar R.A., Thanh C.L., Khatir S., Gandomi A.H. (2023). Revolutionizing sustainable supply chain management: A review of metaheuristics. Eng. Appl. Artif. Intell..

[10] Gorji S.A. (2023). Challenges and opportunities in green hydrogen supply chain through metaheuristic optimization. J. Comput. Des. Eng..

[11] Meraliyev M., Turan C., Kadyrov S., Sadyk U. (2025). A Comprehensive Survey of Methods and Challenges of Vehicle Routing Problem with Uncertainties. Mathematics.

[12] Blum C., Roli A. (2003). Metaheuristics in combinatorial optimization: Overview and conceptual comparison. ACM Comput. Surv..

[13] Talbi E.G. (2009). Metaheuristics: From Design to Implementation.

[14] Pournader M., Ghaderi H., Hassanzadegan A., Fahimnia B. (2021). Artificial intelligence applications in supply chain management. Int. J. Prod. Econ..

[15] Toorajipour R., Sohrabpour V., Nazarpour A., Oghazi P., Fischl M. (2021). Artificial intelligence in supply chain management: A systematic literature review. J. Bus. Res..

[16] Dwivedi A., Jha A., Prajapati D., Sreenu N., Pratap S. (2020). Meta-heuristic algorithms for solving the sustainable agro-food grain supply chain network design problem. Mod. Supply Chain. Res. Appl..

[17] Fahmy S., Gaber Y., Zaki A., Gaafar M. (2024). Fresh produce supply chain network design and management using swarm intelligence: A case study of Egypt. J. Ind. Eng. Manag..

[18] Ghahremani-Nahr J., Nozari H., Szmelter-Jarosz A. (2024). Location-inventory-pricing model in closed-loop supply chain network under the robust-fuzzy optimisation method. Supply Chain Forum: An International Journal.

[19] Pan L., Shan M., Li L. (2023). Optimizing perishable product supply chain network using hybrid metaheuristic algorithms. Sustainability.

[20] Goodarzian F., Kumar V., Abraham A. (2021). Hybrid meta-heuristic algorithms for a supply chain network considering different carbon emission regulations using big data characteristics. Soft Comput..

[21] Chouhan V.K., Khan S.H., Hajiaghaei-Keshteli M. (2021). Metaheuristic approaches to design and address multi-echelon sugarcane closed-loop supply chain network. Soft Comput..

[22] Zhang Y., Wang L. (2024). A Dynamic Scheduling Method for Logistics Supply Chain Based on Adaptive Ant Colony Algorithm. Int. J. Comput. Intell. Syst..

[23] Wang Y., Zhang H., Yuan C., Li X., Jiang Z. (2025). An efficient scheduling method in supply chain logistics based on network flow. Processes.

[24] Şenaras O.M., İnanç Ş., Eren Şenaras A., Öngen Bilir B. (2025). Comparing the Use of Ant Colony Optimization and Genetic Algorithms to Organize Kitting Systems Within Green Supply Chain Management Practices. Sustainability.

[25] Yu J., You X., Liu S. (2022). Dynamically induced clustering ant colony algorithm based on a coevolutionary chain. Knowl.-Based Syst..

[26] Saeed H.A., Al-Janabi S.T.F., Yassen E.T., Aldhaibani O. (2025). A Survey on Secure Scientific Workflow Scheduling in Cloud Environments. Future Internet.

[27] Kundu R., Chattopadhyay S., Nag S., Navarro M.A., Oliva D. (2024). Prism refraction search: A novel physics-based metaheuristic algorithm. J. Supercomput..

[28] Augerat P. (1995). Approche Polyèdrale du Problème de Tournées de Véhicules. Ph.D. Thesis.

